# Combined effects of prohexadione‐calcium and growing environment on sweet cherry fruit quality and postharvest performance

**DOI:** 10.1002/jsfa.70574

**Published:** 2026-03-10

**Authors:** Alice Varaldo, Alessandra Dacomo, Dario Donno, Giovanna Giacalone

**Affiliations:** ^1^ Department of Agricultural, Forest and Food Science DISAFA, University of Turin Grugliasco Italy

**Keywords:** prohexadione‐calcium, growth regulators, nutraceuticals, organic acids, shelf life, sugars, sweet cherry

## Abstract

**BACKGROUND:**

Sweet cherry (*Prunus avium* L.) is a high‐value fruit crop, but its commercial success is often limited by poor pigmentation, reduced firmness, and short shelf life. This study evaluated the effects of preharvest applications of prohexadione‐calcium (1.5 L ha^−1^) on fruit quality and storage performance in lowland and hilly orchards in northern Italy.

**RESULTS:**

Fruit size, weight, firmness, soluble sugars, organic acids, and nutraceutical compounds were measured at harvest and after 14 days of cold storage (2 °C, 75% RH). The firmness of the treated fruits from the hilly orchards was consistently higher and remained stable during storage, while the control fruits experienced an increase in firmness due to dehydration. Prohexadione‐calcium also had an impact on the metabolic composition: in the lowland orchards, the sugars in treated fruits increased, whereas they decreased in the controls, and the opposite trend was observed in the hilly site. The organic acids showed varying responses depending on the site, but generally, the treated fruits retained more tartaric acid and maintained more stable sugar–acid ratios during storage. The nutraceutical qualities were better preserved in the treated fruits, especially at harvest in the hilly site and after 7 days in the lowland site.

**CONCLUSION:**

Prohexadione‐calcium enhanced firmness and nutraceutical stability, improving the postharvest quality of sweet cherries across different growing conditions. © 2026 The Author(s). *Journal of the Science of Food and Agriculture* published by John Wiley & Sons Ltd on behalf of Society of Chemical Industry.

## INTRODUCTION

Sweet cherries (*Prunus avium* L.) are widely cultivated for their taste, nutritional benefits, and economic significance. Europe produces ~31% of the global crop, with Italy standing among the top five European producers and seventh worldwide, averaging 107 000 tons annually.^1^ In recent years, cultivations have spread to less traditional areas like Piedmont, Italy. However, the region faces challenges such as unpredictable spring weather, extreme temperatures, and inconsistent rainfall, all of which can negatively impact both yield and fruit quality. Additionally, the short and weather‐sensitive harvest period makes sweet cherries even more vulnerable.^2^ In Piedmont, the high‐density orchards with dwarfing rootstocks and anti‐hail or rain nets can boost profitability but also create difficulties. The vigorous growth of shoots complicates pruning, which can lead to fewer flower buds and ultimately affect yield and fruit quality, including traits like pigmentation and firmness. While protective nets help prevent hail damage and cracking, they can also have a downside by impacting firmness, a crucial factor for market appeal and shelf life.^3^, ^4^

Plant growth regulators (PGRs) offer promising solutions. Salicylic acid and abscisic acid can improve fruit weight, firmness, phenolic content, and color development,^5^, ^6^, ^7^ while gibberellic acid reduces early fruit drop.^8^ Calcium also helps enhance fruit quality by stabilizing membranes and cell walls.^9^, ^10^, ^11^

Prohexadione‐calcium (Pro‐Ca) is a promising PGR for high‐density orchards, controlling vegetative growth while improving fruit quality. Pro‐Ca inhibits gibberellin biosynthesis, competing with 2‐oxoglutarate at dioxygenases that convert inactive GA20 to active GA1, thus limiting shoot elongation.^12^, ^13^ Its quick absorption (~8 h) and short activity period make it well suitable for the wet spring conditions in Piedmont,^14^ allowing for application during the unpredictable rainy spring seasons. Pro‐Ca has demonstrated efficacy in apples,^15^, ^16^, ^17^, ^18^ pears,^18^, ^19^, ^20^ strawberries,^21^, ^22^, ^23^ and grapes,^24^ with positive aspects on quality and shelf life, improving both growth control and postharvest quality.^13^ However, its use in sweet cherries remains under‐researched, particularly under less favorable climatic conditions. Studies focus on well‐established cherry‐growing regions with optimal climates such as Turkey, Chile, and Spain,^25^, ^26^, ^27^, ^28^, ^29^ leaving significant gaps in understanding Pro‐Ca behavior in regions like Piedmont.

The study reported here evaluated the effects of Pro‐Ca on the quality and shelf life of sweet cherries grown at two altitudes in Piedmont. Specifically, morphological, qualitative, and nutraceutical traits at harvest and during postharvest storage under controlled conditions were assessed. The aim was to investigate Pro‐Ca as a sustainable management strategy to enhance fruit quality, extend shelf life, and provide practical benefits to growers through improved commercial value and reduced postharvest losses.

## MATERIALS AND METHODS

### Plant material and experimental design

The experiment took place during the 2024 growing season in two even‐aged commercial orchards of sweet cherry cv. ‘Sweet Saretta’ in Piedmont, Italy. The trial compared two altitudinal environments. The first site, located in Costigliole Saluzzo (44°34′38″ N, 7°29′45″ E), is at 376 m above sea level, while the second site in Dronero (44°27′48″ N, 7°24′00″ E) is at 622 m above sea level. All trees were planted in 2020, grafted onto ‘Gisela 5’ rootstock and grown under protective netting against rain and hail. Trees were trained to a spindle training system, reaching an average height of 3 m. The lowland site (Costigliole Saluzzo) has a climate with average spring temperatures from 9 to 22 °C, and about 91 mm of total rainfall from March to June. In contrast, the hill site (Dronero) experiences cooler, more variable conditions, with average spring temperatures from 7 to 19 °C, and more rainfall, totaling up to 94 mm during the same period. Furthermore, the differences in daily thermal fluctuations from March to June were greater in the lowland site (13 °C) than the hill site (9 °C).

The experimental design consisted of 6 randomized blocks per site, each composed of 5 trees, for a total of 30 trees per cultivation area (6 × 5 blocks = 30). Three blocks (15 plants) were subjected to foliar treatment (SPRAY), while the remaining three served as an untreated control group (CTRL). The detailed experimental design is shown in the supporting information (Fig. S1).

The foliar treatment was integrated into the farm standard agronomic practices. The treatment involved two applications (Table 1) of the commercial growth regulator REGALIS® (BASF SE, Germany) composed of 10 g of Pro‐Ca and 90 g of co‐formulants per 100 g of product. Harvesting occurred in June, aligned with the local commercial ripening period determined by the characteristic skin color of the ‘Sweet Saretta’ variety. Harvesting was carried out in a single picking, with all fruits collected from each plant. After harvesting, fruits were studied for their quality traits and underwent a 14‐day shelf life evaluation.

**Table 1:** Preharvest agrochemical application for the 2024 season

Phenology stage	BBCH	Operation	Date	
			Lowland	Hill
Petals fall	69	REGALIS® 1.5 L ha^−1^	4 April	19 April
Fruit set	72	REGALIS® 1.5 L ha^−1^	15 April	3 May
Commercial ripeness	89	Harvest	25 June	27 June

### Fruit morphological characteristics

The quality shape was recorded immediately after the harvest, before the transportation to the university laboratory.

#### Plant yield

Yield was recorded for each tree at harvest. Non‐marketable fruits (molded, cracked, deformed, or undersized) were discarded. Marketable fruits were weighed using a field scale, and yield was expressed in kilograms per tree (kg plant^−1^).

#### Fruit caliber

The fruit caliber was determined by measuring the diameter in millimeters (mm), ranging from 26 to 30 mm, with a 2 mm pitch. Measurements were conducted on a sample of 10 kg, containing 900 fruits, per cultivation area and treatment. The fruits were classified into four categories based on their diameter: 26, 28, 30, >30 mm.

#### Fruit weight

The fruit weight was recorded as the weight of 10 cherries per growing site using an electronic scale and then recorded as the individual fruit weight in grams for both CTRL and SPRAY samples. The measurement was carried out ninefold. Results were expressed as single fruit weight (g) by dividing the weight by 10.

### Shelf life test

In both orchards, representative sample batches from each experimental block and treatment were combined and transported to the DISAFA laboratory at the University of Turin (Grugliasco, TO) for quality evaluation. Approximately 20 kg of sweet cherries was randomly selected from each block and carefully checked to ensure uniform quality, i.e. healthiness and absence of defects, before beginning the shelf life test.

For each growing site and treatment, 18 rPET baskets containing 1 kg of cherries were prepared. Furthermore, a subset of nine baskets per treatment and area were analyzed immediately on the harvest day (D0) to establish baseline measurements for the shelf life experiment. The storage trial was conducted over 14 days under controlled conditions of 2 °C and 75% relative humidity in the DISAFA climate chamber (C700BXPRO, FDM s.r.l., Italy).

Throughout the storage period, nine baskets per treatment and growing site were analyzed at two additional time points: the midpoint (D7) and the endpoint (D14). The following parameters were assessed at each time point (D0, D7, and D14): skin color, firmness, total soluble solids (TSS), titratable acidity (TA), sugar profile, organic acid composition, and nutraceutical properties. Analyses on days 7 and 14 also included the evaluation of weight loss (WL) alongside the other quality attributes.

#### Weight loss

Individual samples were weighed using an analytical balance at D0 and each subsequent time point. WL was calculated using Eqn (1), where WL0 represents the basket weight on D0, and WL*x* denotes the weight recorded at a given time point (D7 or D14). The calculated values were expressed as percentage changes relative to the initial weight.

(1) $$\mathrm{WL}=\left(\mathrm{WL}0-\mathrm{WL}x\right)/\mathrm{WL}0$$

#### Skin color

Color measurements were performed using a CR400 colorimeter (Minolta, Singapore, Japan) under consistent lighting conditions with C as standard illuminant and an observation angle of 2°. The measurement was taken on 30 fruits per basket, and the method employed the CIE LAB color space. For the present study results of L*(C) and a*(C) are discussed.

#### Fruit firmness

Fruit firmness was evaluated using a digital durometer (Durofel, Setop Technologie, France). Measurements were taken with a 0.25 cm^2^ probe on 30 fruits per basket under consistent temperature conditions. The results were recorded according to the Durofel firmness scale (0–100).

#### Total soluble solids

TSS was determined using a digital refractometer (ATAGO‐PR‐32, Italy). For the analysis, clear cherry juice was obtained from 10 fruits with a juice extractor and then centrifuged at 1500 × *g* for 10 min using an AVANTIM J‐25 centrifuge (Beckman Instruments, Inc.). The supernatant was collected to perform the analysis. The test was carried out nine times (three replicates per basket), and the results were expressed in °Brix.

#### Titratable acidity and pH

TA was quantified using 10 mL of clear sweet cherry juice, previously extracted and titrated automatically with 0.1 N NaOH to a pH endpoint of 8.1, with an automatic titrator (Titralab AT1000, HACH, France). The analysis was conducted in triplicate for each basket, with results expressed in milliequivalents of NaOH per liter (meq_NaOH_ L^−1^).

#### Determination of sugars and organic acids

In this research, a new method for sugar quantification has been proposed and optimized starting from previously established studies and validated approaches.^30^, ^31^ The preliminary analytical results are promising, but further studies are required to comprehensively evaluate and assess its reproducibility, robustness, and applicability across different matrices and contexts.

##### Extraction protocol

For the extraction of sugars and acids, a protocol has been optimized, varying different parameters (e.g. solute/solvent ratio, extraction times, number of extraction steps, etc.). An amount of 5 g of sweet cherry flesh (collected from 15 fruits) was added to 25 mL of ultrapure water and then dark‐macerated for 30 min. The mixture was homogenized for 1 min using an UltraTurrax T18 basic homogenizer (Janke & Kunkel, IKA®‐Labortechnik, Germany), centrifuged at 3000 × *g* for 15 min, and the resulting supernatant was collected in a flask. The plant material was subjected to a second extraction by adding 25 mL of ultrapure water, vortexing for 30 s, and dark‐macerating for a further 15 min. Following centrifugation, the supernatant was collected again in the flask. This process was repeated a second time to ensure the complete extraction of sugars and acids. A 4 mL aliquot of each extract was filtered through a PTFE filter (25 mm diameter, 0.45 μm) and divided into two 2 mL tubes. One tube was immediately processed for sugar analysis, while the other one was stored at −30 °C until analysis of organic acids. A single extract was prepared for each basket.

##### HPLC analysis

The analysis of sweet cherry extracts was performed using an Agilent 1200 HPLC system (Agilent Technologies, Santa Clara, CA, USA), featuring a G1311A quaternary pump, a manual injector with a sample loop of 20 μL, and a G1315D diode array detector for UV–visible measurements.

Sugar analysis was performed using a SphereClone NH_2_ column (4.6 × 250 mm, 5 μm), and the molecules were detected at a wavelength of 191 nm. The chromatographic conditions consisted of a flow rate of 0.7 mL min^−1^, lasting 30 min with a 2 min post‐run, with a mobile phase composed of ultrapure water as solvent A and acetonitrile as solvent B (35:65 v/v). The gradient analysis consisted of: 65% B to 80% B in 20 min + 80% B to 65% B in 2 min + 65% in 8 min.

Organic acid separation was carried out on a Kinetex C18 column (4.6 × 150 mm, 5 μm), and the components were identified at 214 nm.^32^ The chromatographic conditions included a flow rate of 0.6 mL min^−1^, lasting 13 min with a 2 min post‐run, with 10 mmol L^−1^ KH_2_PO_4_/H_3_PO_4_ solution (pH = 2.8) as mobile phase A and CH₃CN as mobile phase B. The gradient analysis consisted of 5% B to 14% B in 10 min + 14% B in 3 min. More details of chromatographic conditions are presented in the supporting information (Table S1).

Chromatographic peaks were identified by comparing UV–visible spectra and retention times to those of standard solutions of the considered analytes under the same chromatographic conditions. The analysis included four sugar standards (fructose, glucose, sorbitol, and sucrose) and six organic acid standards (quinic acid, succinic acid, malic acid, citric acid, tartaric acid, and oxalic acid). A calibration curve was generated for each standard to determine the relationship between peak area and concentration (Table S1, supporting information). All the measurements were performed ninefold (*N* = 9), and results were expressed as grams per kilogram of fresh weight (mg kg^−1^ f.w.).

#### Nutraceutical composition

##### Extraction protocol

In brief, a mixture of 4 g of cherries (obtained from a representative batch) and 10 mL of extraction solvent (20:1 MeOH–H_2_O acidified with HCl 37%) was homogenized using an UltraTurrax T18 basic (Janke & Kunkel, IKA®‐Labortechnik, Germany) for 1 min. Afterwards, the mixture was sonicated at 50 Hz in a water bath at 50 °C for 20 min and then centrifuged at 1000 × *g* for 10 min.^33^ The supernatant was stored in vials at −26 °C until analysis.

##### Total anthocyanin content

The total anthocyanin content (TAC) was measured using the pH differential method^34^ by diluting a sample with two buffer solutions: pH 1 (0.025 mol L^−1^ KCl) and pH 4.5 (0.4 mol L^−1^ CH_3_COONa). The absorbance of the solutions was measured spectrophotometrically at wavelengths of 520 and 700 nm using a spectrophotometer (U‐5100, Hitachi, Japan). TAC was calculated using the Lambert–Beer law, as outlined in the following formula:

(2) $$\mathrm{TAC}=A\times \mathrm{MW}\times \mathrm{DF}\times \frac{10^{3}}{\varepsilon }\times L$$

where TAC: total anthocyanin content in milligrams of cyanidin‐3‐glucoside per 100 g of fresh cherries (mgCYAN (100 g)^−1^ f.w.); *A*: difference in absorbance (*A*_520nm_ − *A*_700nm_)_pH1_ − (*A*_520nm_ − *A*_700nm_)_pH4.5_; MW: molecular weight of cyanidin (449.2 g mol^−1^); DF: dilution coefficient (10); *L*: optical path in cm; *ε*: extinction coefficient (30.400 L mol^−1^ cm^−1^).

##### Total phenol content

Total phenol content (TPC) was determined using the Folin–Ciocalteu method^35^ with gallic acid as a standard. The absorbance was recorded at 760 nm using a spectrophotometer (U‐5100, Hitachi, Japan). The results were calculated and expressed as milligrams of gallic acid equivalents (GAE) per 100 g of fresh weight (mgGAE (100 g)^−1^ f.w.).

##### Antioxidant capacity

Antioxidant capacity (AOx) was evaluated using the ferric‐reducing antioxidant power (FRAP) assay.^36^, ^37^ The assay is based on the reduction of the Fe^3+^–TPTZ (2,4,6‐tripyridyl‐*s*‐triazine) complex to Fe^2+^ (ferrous) iron at an acidic pH. The reduction of Fe^3+^–TPTZ to Fe^2+^ produces a blue‐colored ferrous tripyridyltriazine complex and was measured spectrophotometrically at 595 nm (U‐5100, Hitachi, Japan) after a 5 min reaction period at 37 °C. The results were expressed as millimoles of Fe^2+^ per kilogram of fresh weight (mmol Fe^3+^ kg^−1^ f.w.).

### Statistical analysis

Data analysis was conducted using R Studio software version 4.1.2 (R Studio, PBC, Boston, MA, USA). A one‐way analysis of variance (ANOVA) was performed to evaluate the effects of treatments (SPRAY and CTRL) on the fruit size and fruit weight, for both cultivation sites with treatment as the fixed factor and ‘block’ as a random factor to account for field variability. For the shelf life test data, a two‐way ANOVA was applied to study the interaction between treatment and time (shelf life timepoint) in both growing areas. *Post hoc* mean comparisons were carried out using Tukey's test, with statistical significance set at *P* ≤ 0.05.

Additionally, correlation analysis was performed to explore relationships among five sugar parameters (fructose, glucose, sorbitol, sucrose, and total sugars) and seven organic acid parameters (citric acid, malic acid, oxalic acid, quinic acid, succinic acid, tartaric acid, and total acids). Data were grouped by treatment (CTRL/SPRAY) and sampling day (D0/D14), and separate analyses were carried out for each combination in both growing environments (Ctrl‐D0, Ctrl‐D14, Spray‐D0, and Spray‐D14). For each subset, a Pearson correlation matrix was computed using only the numeric variables.

## RESULTS

### Fruit morphological characteristics

Pro‐Ca significantly affected fruit caliber (Table 3) and weight (Table 2), with the strongest effects on fruit weight in the hilly area and on fruit size in the lowland site. In the lowland site, SPRAY had no significant effect on fruit weight, whereas in the hilly area, CTRL cherries were heavier. Considering the caliber distribution, there were clear differences between the two environments: lowland cherries were more commonly found in the 26–30 mm categories, whereas hilly cherries were mostly in the >30 mm range. Although SPRAY trees produced higher yields (Table S2), caliber distribution was similar to that of CTRL in the hilly area. In contrast, in the lowland site, SPRAY reduced the proportion of fruits >30 mm, increasing those in the 30 mm class.

**Table 2:** Fruit weight in the SPRAY and CTRL treatments of ‘Sweet Saretta’ sweet cherries during a 14‐day shelf life test. Results are the mean (*n* = 90) ± SE

Treatment	Fruit weight (g)	
	Lowland area	Hill area
CTRL	16.7 ± 0.313	17.5 ± 0.392^a^
SPRAY	16.2 ± 0.283	16.6 ± 0.278^b^
LSD (*P* ≤ 0.05)	ns	*

Groups sharing the same letters are not statistically different, by a one‐way ANOVA. * *p*<0.1; ns, not significant.

**Table 3:** Quantification of cherries in each caliber category (26, 28, 30, >30 mm) on a sample of 900 fruits per treatment

Caliber class	Lowland area		Hill area	
(mm)	CTRL	SPRAY	CTRL	SPRAY
26	0.35%	1.85%	0.00%	0.15%
28	5.03%	10.84%	0.69%	5.04%
30	31.08%	52.22%	20.07%	26.22%
>30	63.54%	35.10%	79.25%	68.59%

**Table 4:** Sugar components in the SPRAY and CTRL treatments of ‘Sweet Saretta’ sweet cherries at harvest (D0) and at the end of shelf life (D14). Results are the mean (*n* = 9) ± SE

Environment	Day	Treatment	Fructose (mg kg^−1^ f.w.)	Glucose (mg kg^−1^ f.w.)	Sorbitol (mg kg^−1^ f.w.)	Sucrose (mg kg^−1^ f.w.)	Total (mg kg^−1^ f.w.)
Hill area	D0	CTRL	267 ± 9^c^	290 ± 17^b^	139 ± 20^b^	189 ± 14^b^	885 ± 53^b^
		SPRAY	204 ± 9^d^	191 ± 20^c^	84 ± 22^c^	124 ± 13^c^	603 ± 55^c^
	D14	CTRL	349 ± 10^a^	426 ± 19^a^	212 ± 20^a^	326 ± 13^a^	1273 ± 55^a^
		SPRAY	301 ± 10^b^	387 ± 22^a^	248 ± 23^a^	306 ± 15^a^	1281 ± 54^a^
	LSD (*P* ≤ 0.05)		*	***	***	*	***
Lowland area	D0	CTRL	321 ± 20^a^	507 ± 21^a^	332 ± 25^a^	458 ± 25^a^	1620 ± 67^a^
		SPRAY	264 ± 19^b^	397 ± 21^b^	159 ± 24^c^	274 ± 24^b^	1090 ± 70^b^
	D14	CTRL	284 ± 20^ab^	299 ± 20^c^	221 ± 27^b^	260 ± 26^b^	1060 ± 64^b^
		SPRAY	334 ± 22^a^	445 ± 21^ab^	345 ± 28^a^	385 ± 25^a^	1510 ± 68^a^
	LSD (*P* ≤ 0.05)		***	***	***	***	***

Groups sharing the same letters are not statistically different, by a two‐way ANOVA (treatment × day). * *p*<0.1, *** *p*<0.001.

**Table 5:** Organic acid components in the SPRAY and CTRL treatments of ‘Sweet Saretta’ sweet cherries at harvest (D0) and at the end of shelf life (D14)

Environment	Day	Treatment	Citric acid (mg kg^−1^ f.w.)	Malic acid (mg kg^−1^ f.w.)	Oxalic acid (mg kg^−1^ f.w.)	Quinic acid (mg kg^−1^ f.w.)	Succinic acid (mg kg^−1^ f.w.)	Tartaric acid (mg kg^−1^ f.w.)	Total (mg kg^−1^ f.w.)
Hill area	D0	CTRL	120.7 ± 13.7a	18.0 ± 1.0a	1.3 ± 0.1a	219.0 ± 15.7a	53.7 ± 2.5a	1.5 ± 0.8c	414.0 ± 29.9a
		SPRAY	51.1 ± 15.0bc	13.9 ± 0.7b	1.1 ± 0.1a	169.0 ± 15.2c	51.3 ± 2.0a	2.5 ± 0.8bc	289.0 ± 34.6b
	D14	CTRL	87.6 ± 14.7ab	18.2 ± 0.7a	3.0 ± 0.1c	184.0 ± 13.4bc	36.0 ± 2.7b	9.6 ± 0.7b	336.0 ± 25.3b
		SPRAY	40.5 ± 15.2c	18.3 ± 0.6a	6.0 ± 0.2b	212.0 ± 20.1ab	55.0 ± 1.9a	14.8 ± 0.9a	341.0 ± 35.3ab
	LSD (*P* ≤ 0.05)		*	**	**	***	***	**	**
Lowland area	D0	CTRL	78.7 ± 6.0ab	16.8 ± 0.4b	0.6 ± 0.1b	260.0 ± 7.5b	41.6 ± 2.0b	21.2 ± 0.8b	419.0 ± 13.0a
		SPRAY	70.9 ± 6.3bc	17.0 ± 0.4b	0.7 ± 0.1b	204.0 ± 7.4c	45.7 ± 2.2ab	26.3 ± 0.7a	365.0 ± 12.2b
	D14	CTRL	54.6 ± 6.7c	17.8 ± 0.5b	0.8 ± 0.1b	177.0 ± 7.3d	49.2 ± 2.9a	6.8 ± 0.8d	306.0 ± 19.8c
		SPRAY	98.0 ± 6.3a	25.1 ± 0.5a	1.1 ± 0.1a	287.0 ± 7.9a	26.7 ± 3.0c	17.2 ± 0.8c	455.0 ± 12.4a
	LSD (*P* ≤ 0.05)		***	***	*	***	***	**	***

Results are the mean (*n* = 9) ± SE. Groups sharing the same letters are not statistically different, by a two‐way ANOVA (treatment × day). * *p*<0.1, ** *p*<0.01, *** *p*<0.001.

### Shelf life assessment

To provide a more comprehensive presentation of the results, the qualitative parameters of the shelf life test are reported based on the statistical interaction between the Pro‐Ca treatment and shelf life period (treatment × timepoint) across both cultivation areas.

#### Mechanical properties

**Figure 1:**
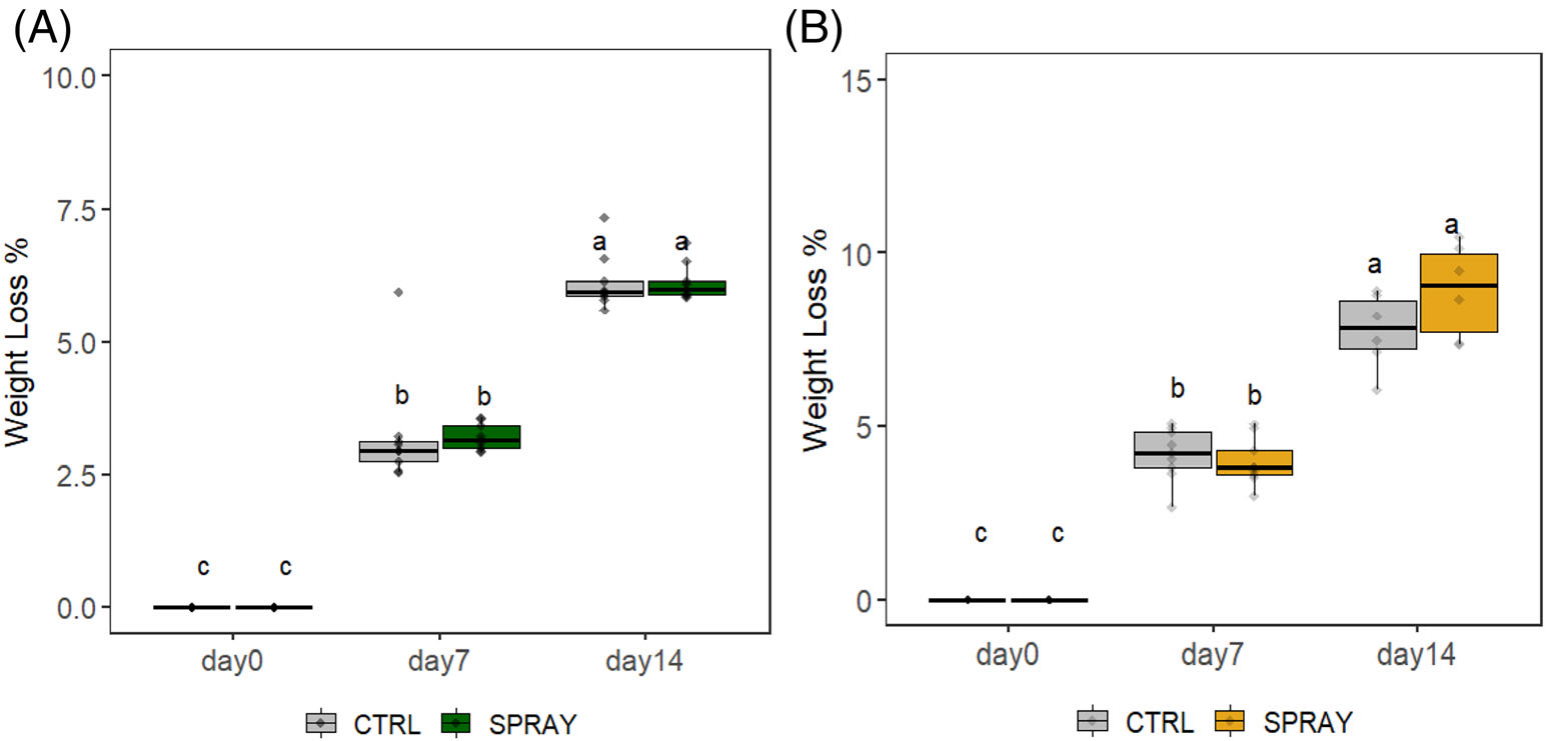
WL development of ‘Sweet Saretta’ sweet cherries during a 14‐day shelf life test. (A) Hill growing area (*n* = 9). (B) Lowland growing area (*n* = 9). Groups sharing the same letters are not statistically different, by a two‐way ANOVA (treatment × timepoint). Statistical significance at *P* < 0.01, by the Tukey LSD test.

Pro‐Ca did not affect fruit WL in either environment (Fig. 1). WL increased during storage, reaching the highest values on D14, and was greater in lowland than hilly cherries. No treatment effects were observed.

**Figure 2:**
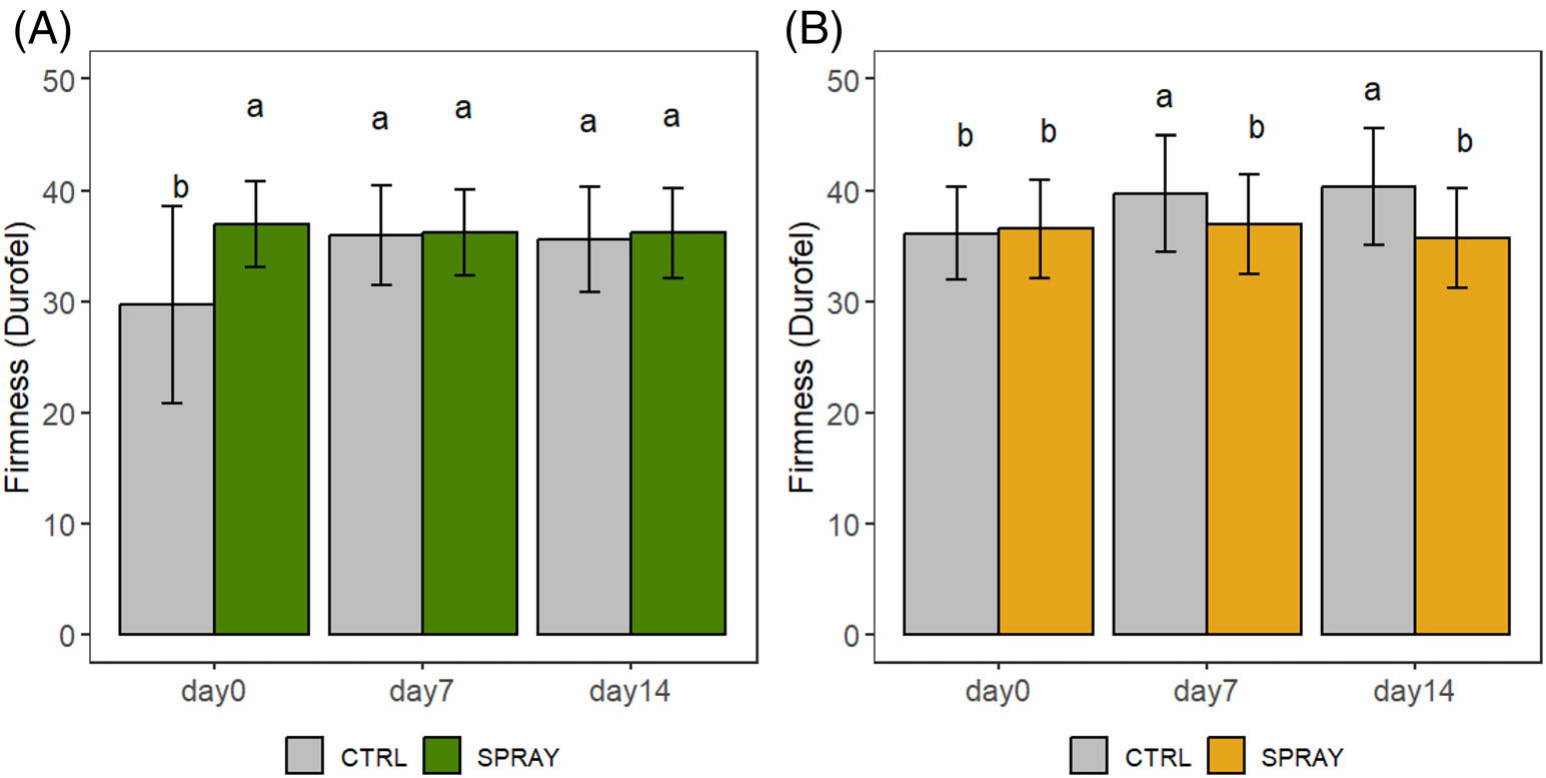
Firmness development of ‘Sweet Saretta’ sweet cherries during a 14‐day shelf life test. (A) Hill growing area (*n* = 60). (B) Lowland growing area (*n* = 60). Groups sharing the same letters are not statistically different, by a two‐way ANOVA (treatment × timepoint). Statistical significance at *P* < 0.01, by the Tukey LSD test.

Firmness patterns differed from those of WL (Fig. 2). In the hilly site (Fig. 2(A)), CTRL cherries were softer at harvest and became firmer during storage, while SPRAY fruits maintained similar firmness throughout. A similar trend was observed in the lowland site (Fig. 2(B)): SPRAY cherries maintained firmness, whereas CTRL cherries increased from the lowest values at harvest to the highest on D14. Overall, Pro‐Ca treatment helped maintain firmness, especially in hilly conditions.

#### Skin pigmentation

**Figure 3:**
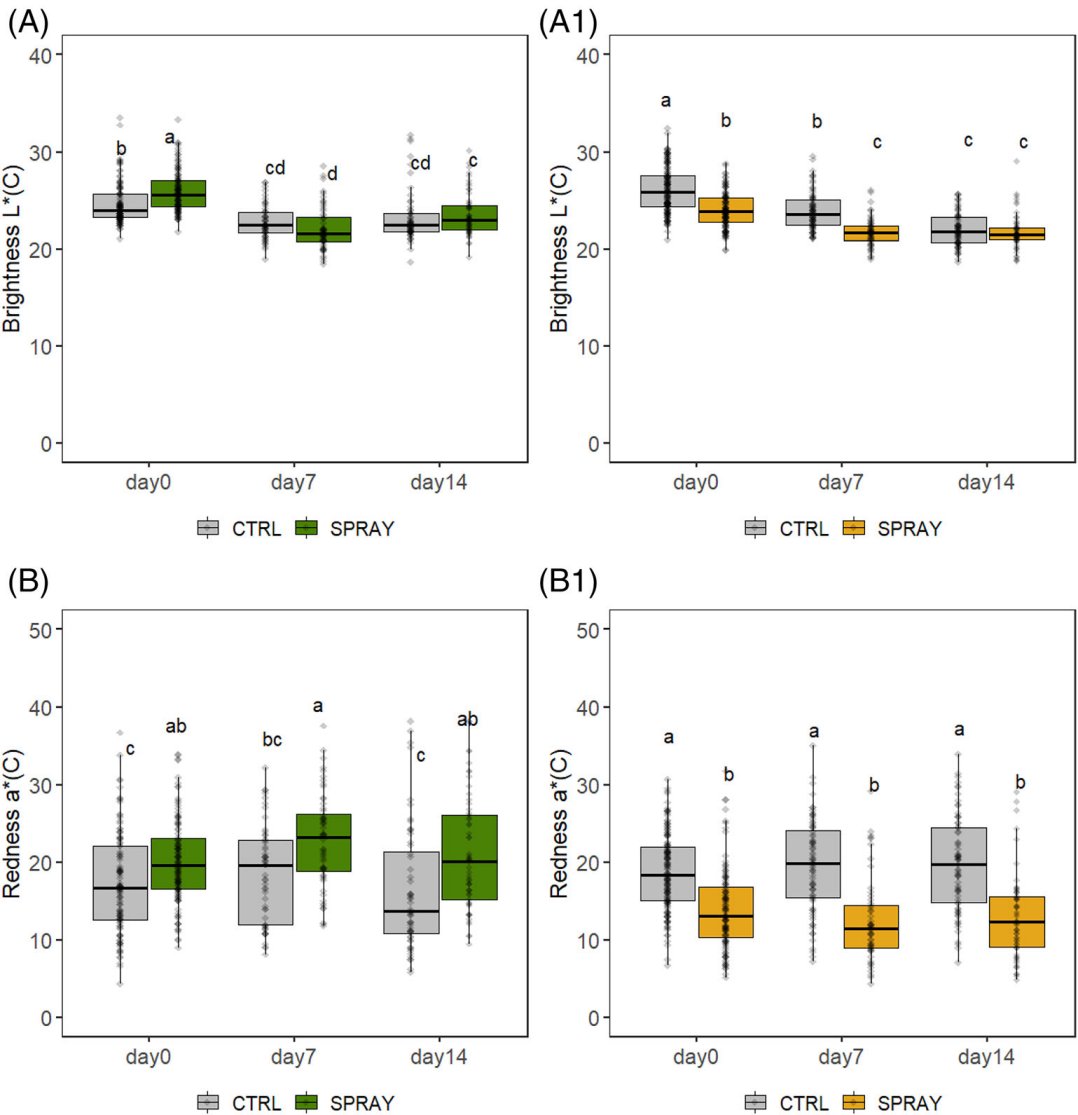
Skin color of ‘Sweet Saretta’ sweet cherries during a 14‐day shelf life test. (A) Brightness from the hill growing area L*(C) (*n* = 90). (A1) Brightness from the lowland growing area L*(C) (*n* = 90). (B) Redness from the hill growing area a*(C) (*n* = 90). (B1) Redness from the lowland growing area a*(C) (*n* = 90). Groups sharing the same letters are not statistically different, by a two‐way ANOVA (treatment × time point). Statistical significance at *P* < 0.0001 for (A, A1, B) and *P* < 0.001 for (B1) by the Tukey LSD test.

Fruit color dynamics are shown in Fig. 3. Brightness (L*) decreased during storage in both environments. In the hilly site, SPRAY cherries were significantly brighter on D0, while in the lowland site, CTRL fruits were brighter. By D14, no differences were observed between treatments.

Red pigmentation (a*) increased in SPRAY cherries from the hilly site, reaching the highest values on D7, whereas CTRL cherries had the lowest pigmentation on D0 and D14. In the lowland site, the opposite trend was recorded: CTRL fruits exhibited significantly higher redness across all time points.

#### Sugars and organic acids

##### Total sugar and acid content

**Figure 4:**
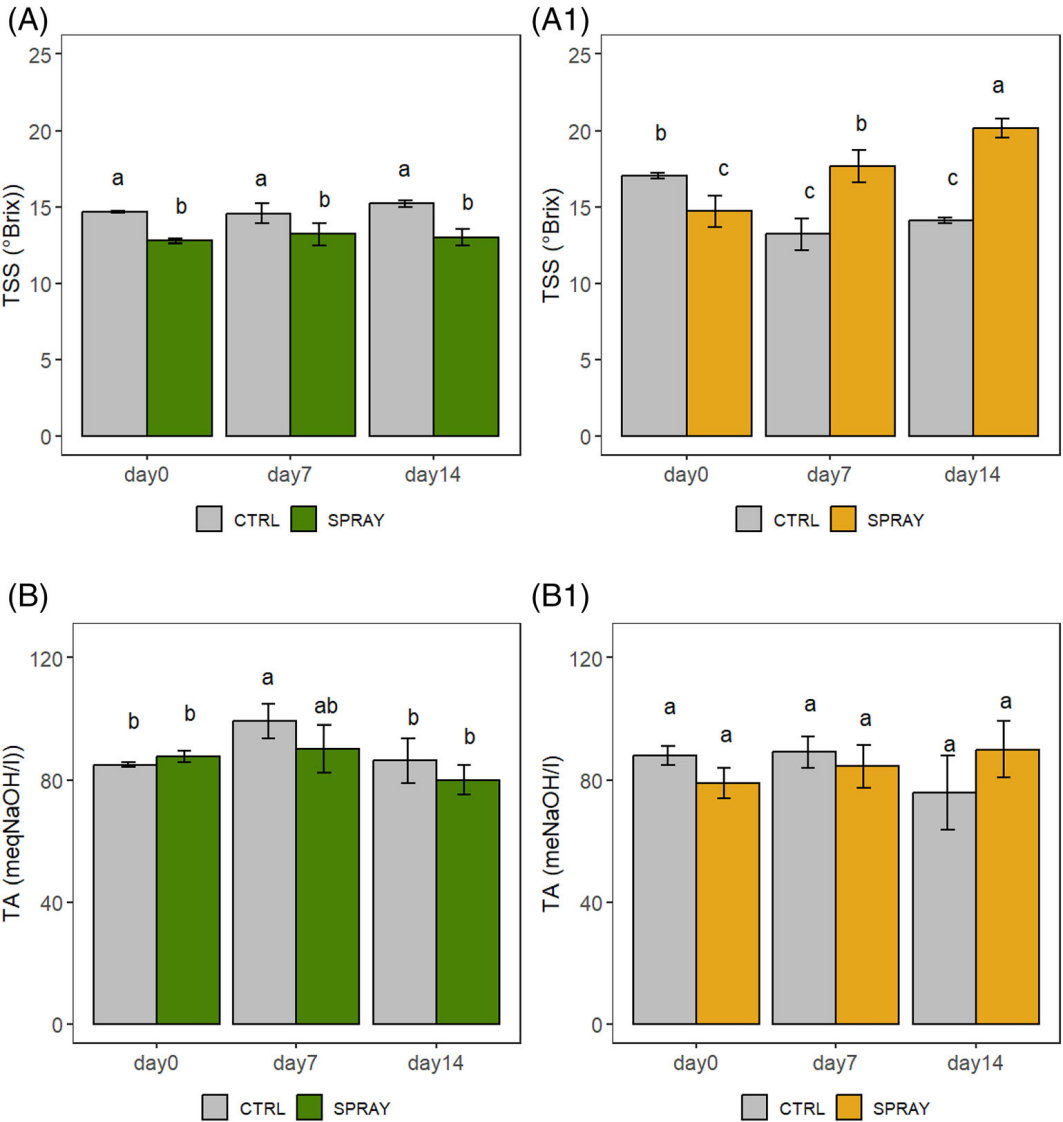
TSS and TA of ‘Sweet Saretta’ sweet cherries during a 14‐day shelf life test. (A) TSS from the hill growing area (*n* = 9). (A1) TSS from the lowland growing area (*n* = 9). (B) TA from the hill growing area (*n* = 9). (B1) TA from the lowland growing area (*n* = 9). Groups sharing the same letters are not statistically different, by a two‐way ANOVA (treatment × timepoint). Statistical significance at *P* < 0.001 by the Tukey LSD test.

The TSS and TA are presented in Fig. 4. The sugar concentration of SPRAY cherries grown in the hill area (Fig. 4(A)) displayed lower levels than CTRL throughout the entire storage period, without statistically significant differences among time points. In contrast, the pattern observed in the lowland site (Fig 4(A1)) was different: the highest sugar concentration was recorded in SPRAY fruits on D14, while the lowest levels were found also in SPRAY cherries but on D0 and in CTRL fruits on D7 and D14. Overall, sugar content remained relatively stable during storage for cherries grown in the hill area. Conversely, while Pro‐Ca‐treated lowland cherries showed an increasing trend in sugar content, untreated lowland cherries exhibited a decreasing trend over time.

In terms of total acid content, the trend remained relatively stable during the storage period. In the hill area (Fig. 4(B)), CTRL fruits on D7 showed the highest TA, while both CTRL and SPRAY fruits exhibited the lowest acid concentrations on D0 and D14. In the lowland area (Fig. 4(B1)), no statistically significant differences in TA were observed between treatments or among the storage time points.

##### Composition of sugars and organic acids

To gain a deeper understanding of sugar and acid development during the shelf life test, a fingerprinting analysis of sugars and organic acids was performed at the beginning (D0) and at the end (D14) of storage. In the cherry sugar profiling (Table 4), the following components were identified: fructose, glucose, sorbitol, and sucrose. The organic acids detected (Table 5) were citric, malic, oxalic, quinic, succinic, and tartaric acids.

In the hilly site, all sugars increased during storage, with higher initial levels in CTRL. By D14, differences between treatments disappeared for most sugars, except for fructose, which remained higher in CTRL. In the lowland site, CTRL cherries had higher sugars at harvest, but SPRAY cherries surpassed them by D14.

Acid profiles also experienced a variation. Citric and quinic acids were the most abundant. In the hilly site, CTRL cherries started with higher levels that declined during storage, while those of SPRAY cherries increased. Similar patterns occurred in the lowland site, where CTRL decreased and SPRAY increased. Malic and quinic acids tended to increase in SPRAY cherries but decreased in CTRL. Tartaric and succinic acids showed site‐ and treatment‐dependent variations.

Correlation analysis (Figs S2 and S3) revealed distinct patterns between sugars and organic acids depending on treatment, storage time, and growing environment. At harvest, CTRL fruits showed strong positive correlations among individual sugar components in both environments (Figs S2(A) and S3(A)), while Pro‐Ca‐treated fruits exhibited a weaker or even negative relationship, particularly if considering fructose (Figs S2(B) and S3(B)). Furthermore, CTRL‐D0 from hillside presents a total positive correlation between all components except for oxalic acid, while for lowland, this pattern is not observed, and a strong negative correlation of malic acid and quinic acid with all the sugars is noted.

After 14 days of storage, Pro‐Ca‐treated fruits showed positive correlations between sugars and several organic acids, particularly citric, malic, and quinic acids (Figs S2(B1) and S3(B1)). In contrast, a strong negative correlation between tartaric and succinic acids and all other components was observed in the lowland environment, while only tartaric acid showed this pattern in the hilly site. In contrast, CTRL fruits exhibited more variable patterns. Fructose and glucose are negatively correlated with acids in both environments except for tartaric acid, which is positively correlated in hill conditions.

Overall, Pro‐Ca treatment appeared to reduce the differences between growing environments. Correlation patterns in SPRAY‐D0 fruits were similar between hill and lowland sites, a trend largely maintained after storage, except for succinic acid. In contrast, such convergence was not observed in CTRL‐D0 fruits. However, postharvest storage partially minimized these differences, as CTRL‐D14 fruits from both environments showed comparable patterns, although less marked than SPRAY fruits.

##### Nutraceutical composition

**Figure 5:**
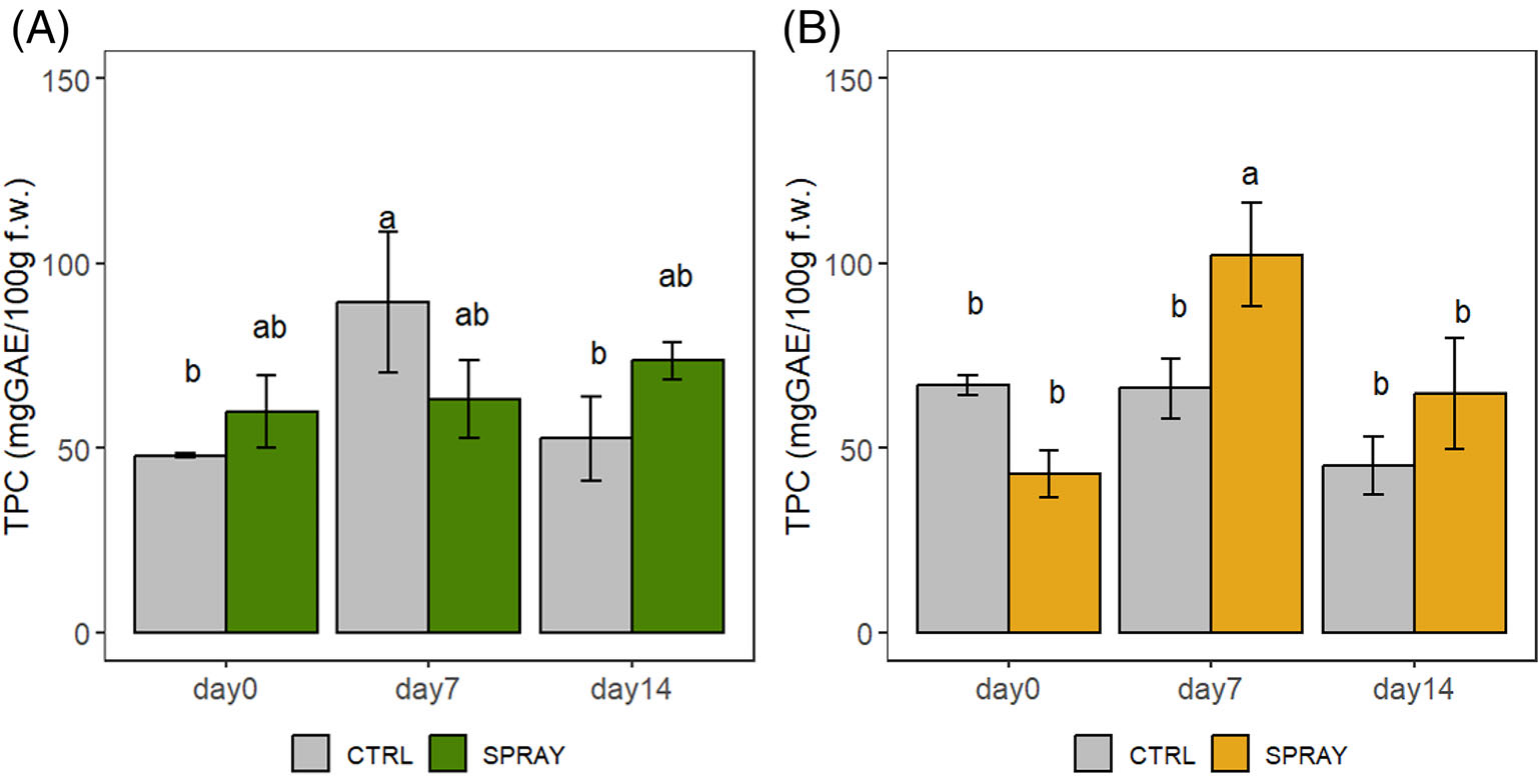
TPC of ‘Sweet Saretta’ sweet cherries during a 14‐day shelf life test. (A) Hill growing area (*n* = 9). (B) Lowland growing area (*n* = 9). Groups sharing the same letters are not statistically different, by a two‐way ANOVA (treatment × timepoint). Statistical significance at *P* < 0.001, by the Tukey LSD test.

TPC was affected by treatment and site (Fig. 5). In the hilly site, SPRAY cherries had higher TPC at harvest, although not significantly so, while in the lowland, no differences were found at D0. During storage, intermediate variations were observed, but no consistent trend emerged.

**Figure 6:**
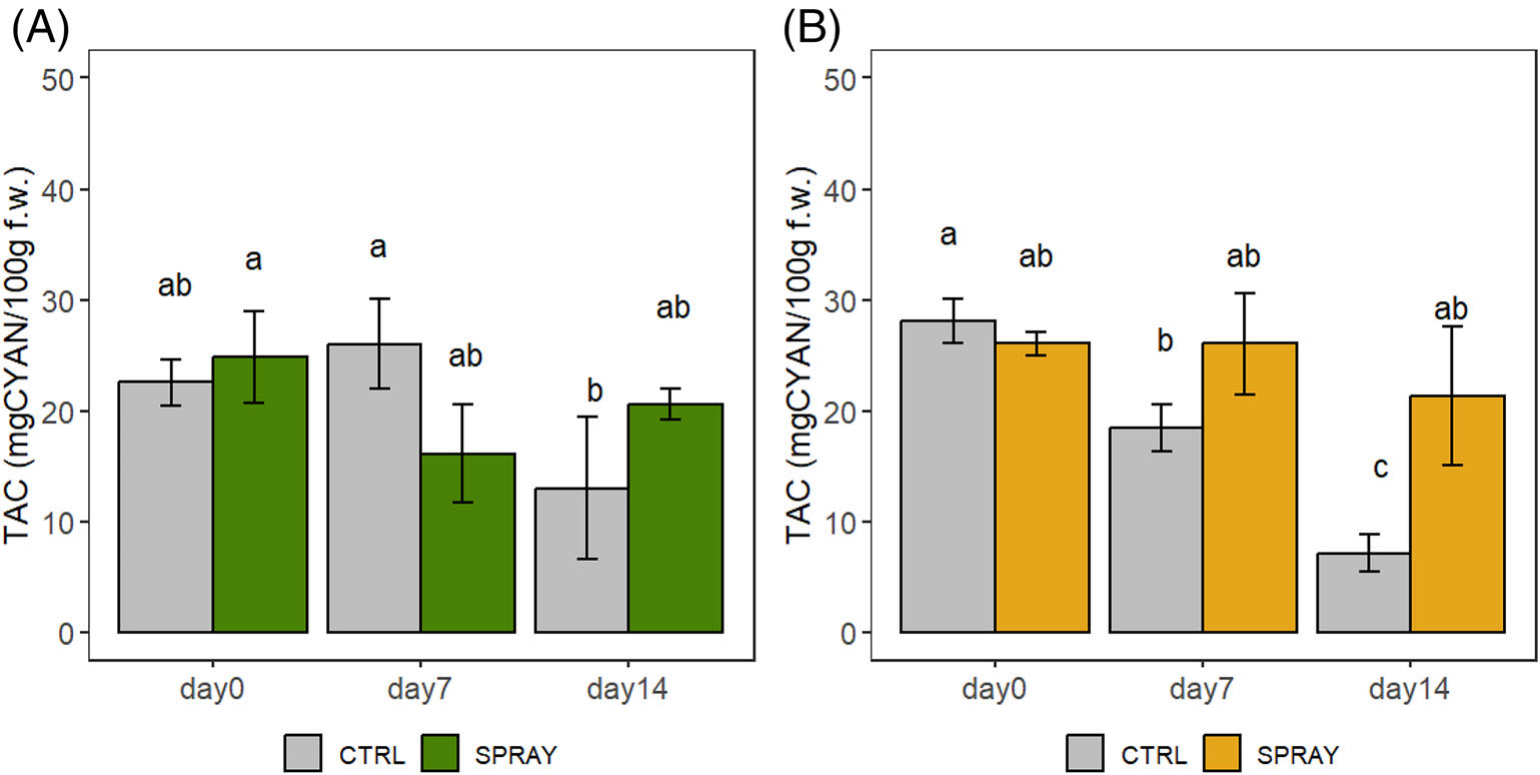
TAC of ‘Sweet Saretta’ sweet cherries during a 14‐day shelf life test. (A) Hill growing area (*n* = 9). (B) Lowland growing area (*n* = 9). Groups sharing the same letters are not statistically different, by a two‐way ANOVA (treatment × timepoint). Statistical significance at *P* < 0.001, by the Tukey LSD test.

TAC (Fig. 6) showed similar dynamics. On the hilly site, SPRAY cherries had higher concentrations at harvest, although not significantly, while CTRL peaked at D7 but significantly declined by D14. In the lowland site, CTRL cherries decreased significantly during storage, while SPRAY maintained the same concentration from D0 to D14.

**Figure 7:**
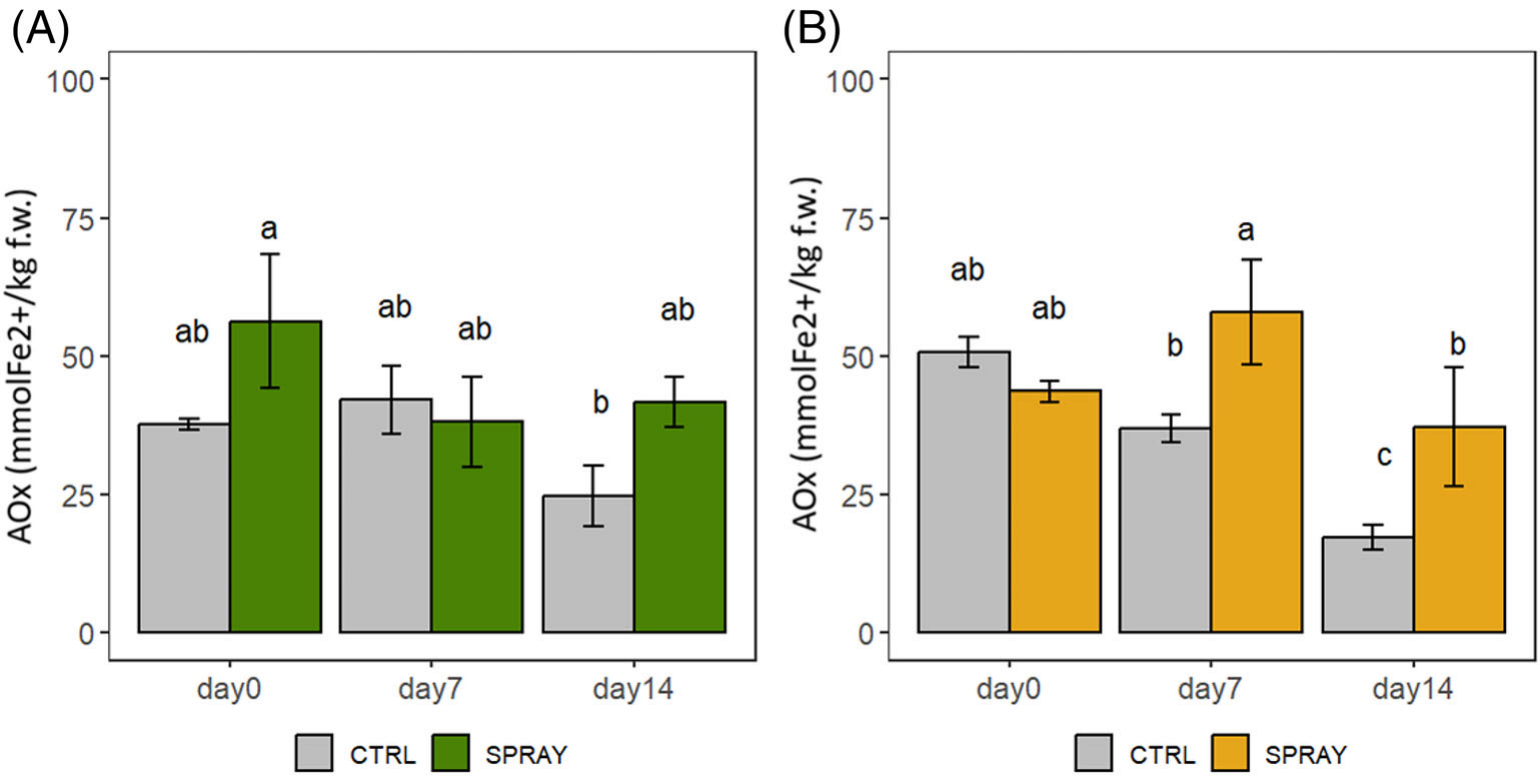
AOx of ‘Sweet Saretta’ sweet cherries during a 14‐day shelf‐life test. (A) Hill growing area (*n* = 9). (B) Lowland growing area (*n* = 9). Groups sharing the same letters are not statistically different, by a two‐way ANOVA (treatment × timepoint). Statistical significance at *P* < 0.001, by the Tukey LSD test.

AOx (Fig. 7) followed the TPC trend. SPRAY and CTRL cherries maintained stable AOx across storage, especially in the lowland site. In hill conditions, SPRAY fruits preserved AOx during storage, while CTRL decreased their concentration significantly at D14.

In general, Pro‐Ca treatment supported greater stability of phenolic compounds, anthocyanins, and antioxidant activity during storage.

## DISCUSSION

### Fruit morphological characteristics

Fruit traits at harvest were influenced by both Pro‐Ca application and cultivation area. In the lowland orchard, SPRAY fruits were slightly smaller, grouping around 30 mm, whereas a greater proportion of CTRL fruits exceeded this size. This trend may be related to higher yield in SPRAY trees and the inhibitory effect of Pro‐Ca on gibberellin biosynthesis, known to limit cell expansion.^25^, ^38^ Nevertheless, SPRAY fruits remained within the highly marketable caliber. Recent data from Italian markets highlight strong consumer demand for cherries between 28 and 30 mm in diameter.^39^ In the hillside orchard, average weight was higher in CTRL fruits, but size distribution was similar between treatments, suggesting that environmental conditions such as reduced vigor and slower metabolism may buffer the effects of the growth regulator.

### Mechanical properties development

WL during storage was mainly affected by the cultivation site, with hillside cherries showing lower WL. This effect may be attributed to microclimatic factors typical of hilly areas (e.g. lower temperatures, reduced solar radiation, higher diurnal temperature variation, and thicker cuticles), which can reduce transpiration and slow the degradation of key metabolites, ultimately improving postharvest quality.^38^, ^40^, ^41^, ^42^

Interestingly, these findings appear to contrast with the established role of Ca in reinforcing cell walls and stabilizing the cuticle, thereby reducing water permeability.^43^

Firmness was more clearly influenced by treatment. At harvest, hillside SPRAY fruits were significantly firmer than CTRL, and firmness was maintained during storage in both sites. Similar results were reported by Correia *et al*.,^44^ who found that calcium‐based treatments preserved fruit consistency during shelf life. In contrast, CTRL fruits exhibited an apparent firmness increase during storage, which does not reflect a true improvement of fruit texture. Since WL did not differ significantly between treatments, this behavior is unlikely to be driven by dehydration‐induced tissue stiffening.^45^, ^46^ Instead, changes in osmotic balance, possibly associated with higher glucose and fructose concentrations measured in CTRL fruits at D14, may have contributed to increased tissue resistance to penetration.^47^ Moreover, lower oxalic acid levels in CTRL fruits could have supported greater Ca^2+^ availability by limiting calcium oxalate formation, thereby favoring cell wall stability.^48^, ^49^

### Sugar and organic acid composition

Glucose and fructose are the main sugars of sweet cherries (*ca* 90% of the totals), crucial for sensory quality.^50^, ^51^, ^52^ TSS readings do not align with total sugar, likely due to refractometers measuring other refractive substances, such as acids, amino acids, proteins, minerals, and polyphenols, beyond sugars.^53^ In sweet cherries, the high anthocyanin concentrations can raise °Brix readings to 32%.^54^

At harvest, SPRAY fruits showed lower sugars in both areas. During storage, dehydration‐related concentration processes occurred similarly in all samples, as indicated by comparable WL. Under these conditions, sugar concentration alone would be expected to be balanced between treatments. However, lowland CTRL cherries exhibited a net decrease in sugars, consistent with a higher respiratory activity that likely exceeded the transpiration‐driven concentration effect. In contrast, in hill CTRL fruits, the concentration effect appeared to prevail, resulting in increased sugar levels. Although Pro‐Ca can improve photosynthetic efficiency,^16^, ^19^, ^22^ these effects may be diminished under high fruit loads. Overall, the contrasting sugar trends observed between hill and lowland environments likely reflect differences in temperature‐driven respiratory activity, which in lowland conditions appeared to be partially mitigated by Pro‐Ca treatment.

Organic acids contribute substantially to quality and are closely interconnected to sugar metabolism.^54^, ^55^, ^56^ In the present study, acid levels largely reflected sugar trends, often being lower in SPRAY fruits, in line with what was reported by Ağlar.^25^ Unlike sugars, the major organic acids showed a similar trend across both growing environments and treatments, suggesting that their postharvest evolution is mainly governed by intrinsic metabolic processes during storage, such as respiration‐related pathways and acid interconversion, rather than by preharvest environmental conditions.

SPRAY cherries generally retained higher tartaric acid, which is not metabolized in the tricarboxylic acid cycle and is often associated with lower respiration rates.^26^, ^57^ Malic acid levels in SPRAY fruits, particularly in hillside conditions, may also be linked to reduced cracking, as malic acid can influence membrane permeability.^58^ Correlation analysis indicated a negative relationship between sugars and acids in lowland CTRL fruits, reflecting stress‐driven shifts in carbon metabolism,^59^ whereas hill‐grown cherries displayed more balanced profiles, as also reported in other temperate crops.^60^

Organic acid behavior, particularly in relation to sugars, varied significantly between hill and lowland areas, reflecting the influence of altitude and temperature known to accelerate respiratory activity, as well as better organization of metabolites due to a slower fruit maturation.^41^, ^61^, ^62^ Notably, Pro‐Ca reduced the divergence between environments, stabilizing sugar–acid interactions. The marked negative correlation between succinic acid and other metabolites in lowland SPRAY fruits may indicate activation of alternative respiratory pathways, such as GABA or AOX, in response to combined heat stress and Pro‐Ca‐induced hormonal changes.^12^, ^63^, ^64^, ^65^

### Fruit pigmentation and nutraceutical composition

Color development was also affected by Pro‐Ca and the environment. In hillside orchards, SPRAY fruits displayed more intense coloration, possibly due to reduced canopy and higher thermal amplitude. Pro‐Ca may reduce anthocyanin biosynthesis via gibberellin inhibition, yet in our study treated fruits retained anthocyanins better during storage, likely supported by tartaric acid involvement in pH stabilization.^63^, ^64^ In contrast, in the lowland site, CTRL fruits exhibited higher a* values than SPRAY fruits. This opposite response may be related to differences in crop load and ripening dynamics, as Pro‐Ca‐treated trees in the lowland environment showed higher yield, which may have slightly delayed color development at harvest.

Nutraceutical parameters (TAC, TPC, AOx) followed similar trends, with SPRAY fruits showing improved preservation during storage. This agrees with findings in strawberries,^21^ apples,^15^ pears,^20^ and horticultural crops,^65^ where Pro‐Ca preserved phenolic stability. Higher quinic acid in SPRAY fruits at the end of storage further supports a role in phenolic metabolism and quality retention, consistent with observations by Zhang and Whiting.^63^

## CONCLUSIONS

Preharvest application of Pro‐Ca improved multiple quality traits of sweet cherries cv. ‘Sweet Saretta’, with effects varying by environment. Its impact was most consistent in hillside orchards, enhancing fruit weight, firmness, and red pigmentation, while in lowland orchards benefits were mainly seen in sugar–acid balance and size distribution. Across both environments, Pro‐Ca preserved firmness, anthocyanins, phenolics, and antioxidant capacity during storage, and reduced respiration rate, supporting better postharvest quality. These results highlight that the effectiveness of Pro‐Ca depends on environmental conditions, suggesting its use is particularly advantageous in hilly orchards. Further studies using targeted analytical methods and gene expression analyses are needed to clarify underlying biochemical pathways and extend findings to other cultivars.

## AUTHOR CONTRIBUTIONS

GG: Conceptualization, supervision, project administration. AV, AD and DD: Data curation, formal analysis. AV and AD: Investigation, methodology. GG and DD: validation, writing – review and editing. AV: Writing – original draft.

## Supporting information


**Table S1.** Chromatographic conditions for sugars and organic acids methods.
**Table S2.** Plant production in the SPRAY and control group (CTRL) treatments of ‘Sweet Saretta’ sweet cherries during a 14‐days shelf life. Results are the mean (*n* = 15) ± SE. Groups sharing the same letters are not statistically different, by one‐way ANOVA.
**Figure S1.** Experimental design. (A) Hill environment (green blocks), (B) lowland environment (yellow blocks).
**Figure S2.** Correlation matrix between sugar and acid components of ‘Sweet Saretta’ sweet cherries grown in a hill environment on the day of the harvest (D0) and at the end of the shelf life (D14).
**Figure S3.** Correlation matrix between sugar and acid components of ‘Sweet Saretta’ sweet cherries grown in a lowland environment at the day of the harvest (D0) and the end of the shelf life (D14).

## Data Availability

The data that support the findings of this study are available from the corresponding author upon reasonable request.
