# Supplementary material for: Combined effects of prohexadione‐calcium and growing environment on sweet cherry fruit quality and postharvest performance
Source: J Sci Food Agric. 2026 Mar 10;106(8):4901–12. doi: 10.1002/jsfa.70574 (PMC13157241; doi:10.1002/jsfa.70574)
Supplement: Supplementary file 1 — Table S1. Chromatographic conditions for sugars and organic acids methods. Table S2. Plant production in the SPRAY and control group (CTRL) treatments of ‘Sweet Saretta’ sweet cherries during a 14‐days shelf life. Results are the mean (n = 15) ± SE. Groups sharing the same letters are not statistically different, by one‐way ANOVA. Figure S1. Experimental design. (A) Hill environment (green blocks), (B) lowland environment (yellow blocks). Figure S2. Correlation matrix between sugar and acid components of ‘Sweet Saretta’ sweet cherries grown in a hill environment on the day of the harvest (D0) and at the end of the shelf life (D14). Figure S3. Correlation matrix between sugar and acid components of ‘Sweet Saretta’ sweet cherries grown in a lowland environment at the day of the harvest (D0) and the end of the shelf life (D14). [file JSFA-106-4901-s001.docx]

**Table S 1.** Chromatographic conditions for sugars and organic acids methods

| **Class** | **Standard** | **Wavelenght** | **Calibration curve equation** | **R^2^** | **Calibration curve range** |
| --- | --- | --- | --- | --- | --- |
|  |  | (nm) |  |  | (mg/l) |
| Sugars | fructose | 191 | y = 1.2199x - 16.928 | 0.999 | 125 – 1000 |
|  | glucose | 191 | y = 0.3712x + 54.368 | 0.999 | 125 – 1000 |
|  | sucrose | 191 | y = 0.1136x + 10.558 | 0.998 | 250 – 2000 |
|  | sorbitol | 191 | y = 0.5625x + 12.363 | 0.998 | 62.5 – 1000 |
|  |  |  |  |  |  |
| Organic acids | citric acid | 214 | y = 1.0603x - 22.092 | 1.000 | 167 – 1000 |
|  | malic acid | 214 | y = 1.4150x - 80.254 | 0.996 | 167 – 1000 |
|  | oxalic acid | 214 | y = 6.4502x + 6.1503 | 0.998 | 167 – 1000 |
|  | quinic acid | 214 | y = 0.8087x - 38.021 | 0.998 | 167 – 1000 |
|  | succinic acid | 214 | y = 0.9236x - 8.082 | 0.995 | 167 – 1000 |
|  | tartaric acid | 214 | y = 1.8427x + 15.796 | 1.000 | 167 – 1000 |

**Table S 2.** Plant production in the SPRAY and CTRL treatments of ‘Sweet Saretta’ sweet cherries during a 14-days shelf life. Results are the mean (n=15) ± SE. Groups sharing the same letters are not statistically different, by one-way ANOVA.

| Treatment | Plant Production (kg) | | | | | | | | |
| --- | --- | --- | --- | --- | --- | --- | --- | --- | --- |
|  | Lowland area | | | |  | Hill area | | | |
| CTRL | 2.92 | ± | 0.47 | b |  | 1.58 | ± | 0.31 | b |
| SPRAY | 6.42 | ± | 0.40 | a |  | 3.88 | ± | 0.25 | a |
| LSD p≤0.05 | *** | | | |  | *** | | | |

***Figure S 1.*** *Experimental design. A: Hill environment (green blocks), B: Lowland environment (yellow blocks)*


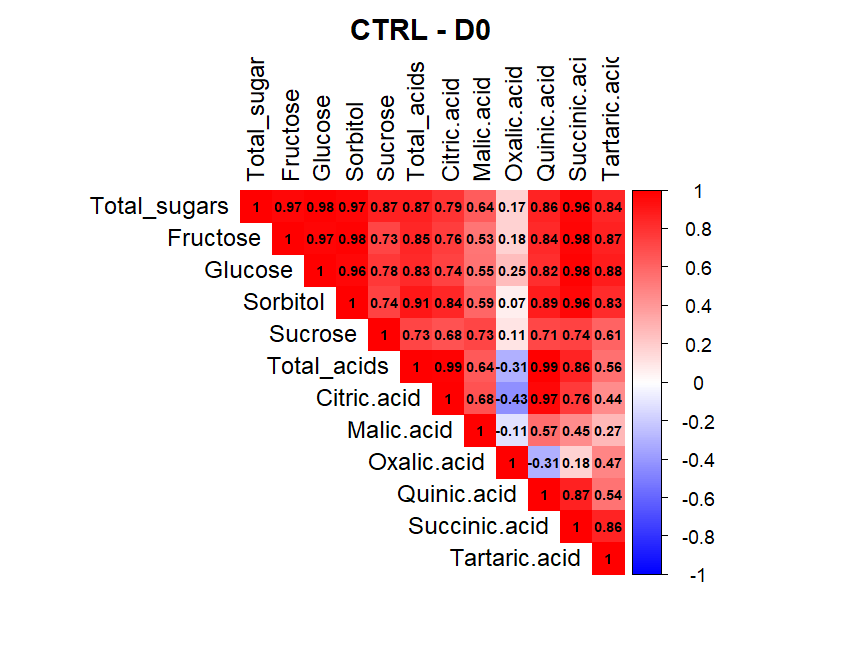

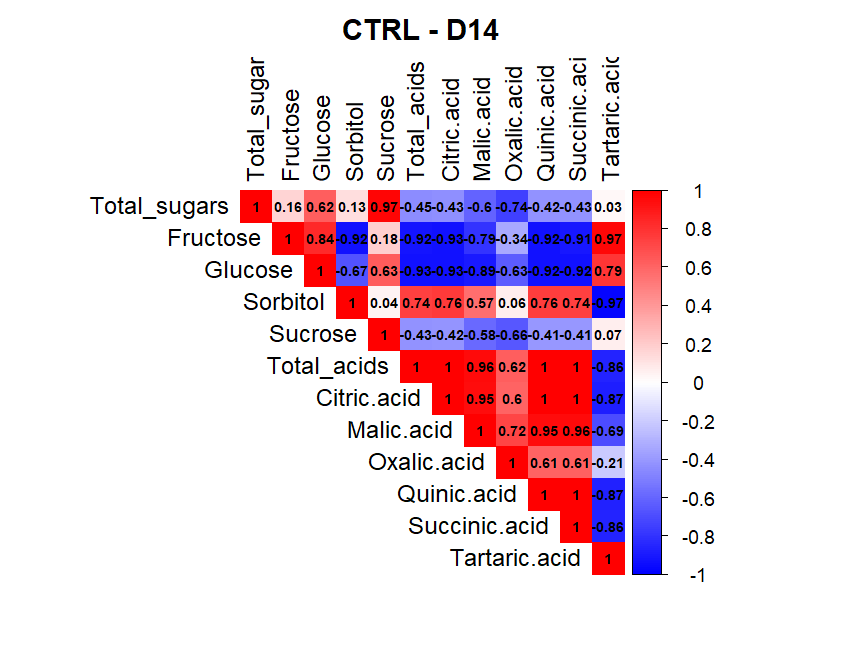

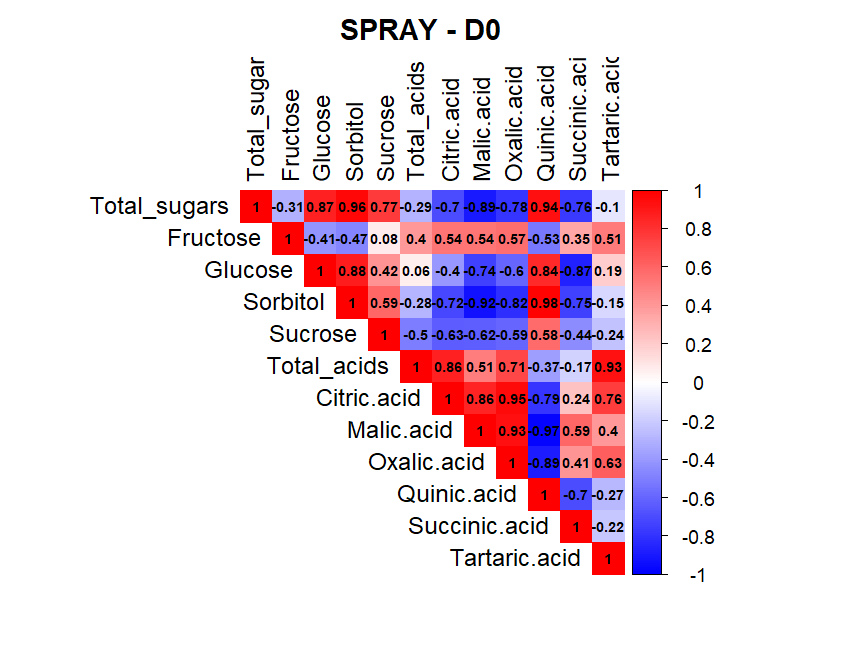

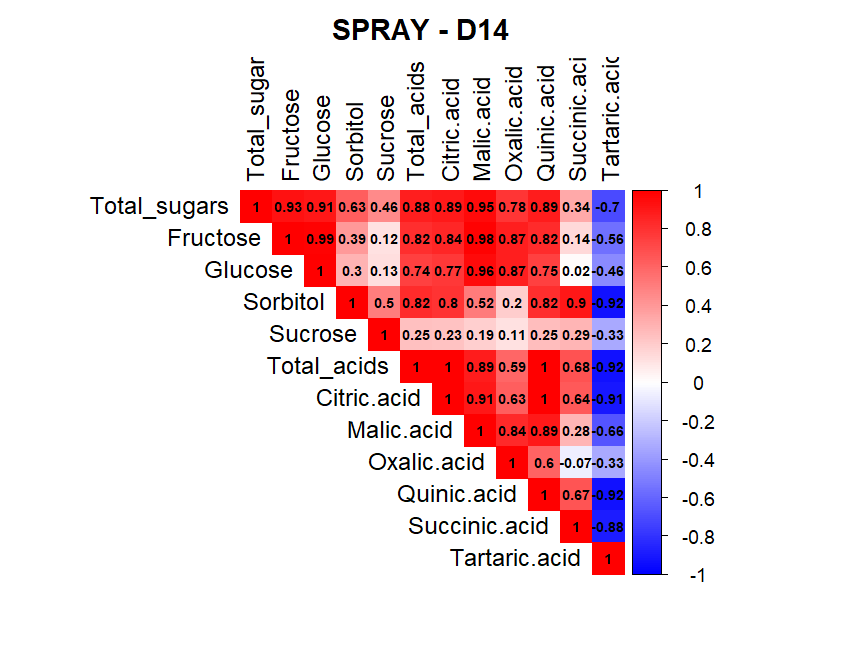


B

B1

A1

A

**Figure S 2**. Correlation matrix between sugar and acid components of ‘Sweet Saretta’ sweet cherries grown in a hill environment on the day of the harvest (D0) and at the end of the shelf life (D14).


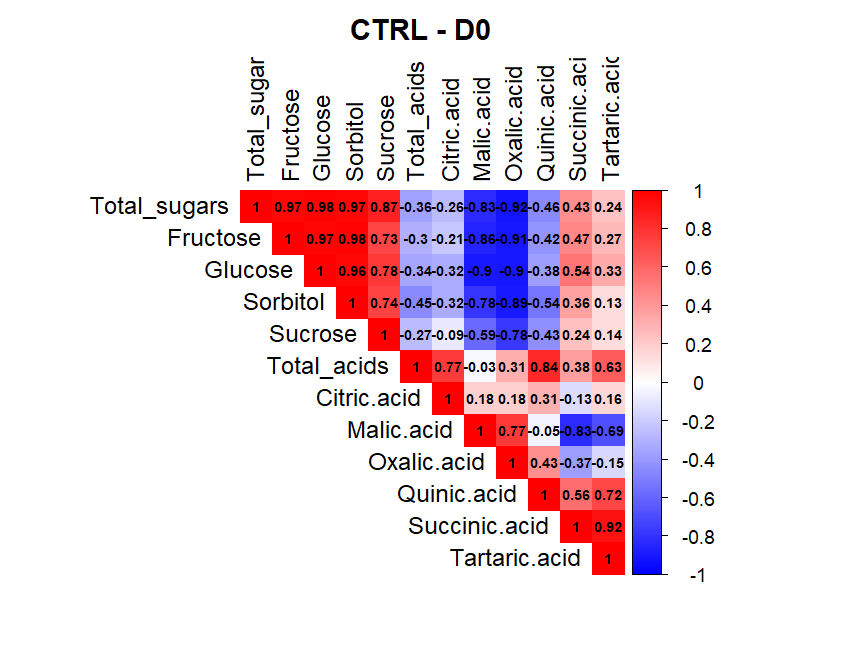

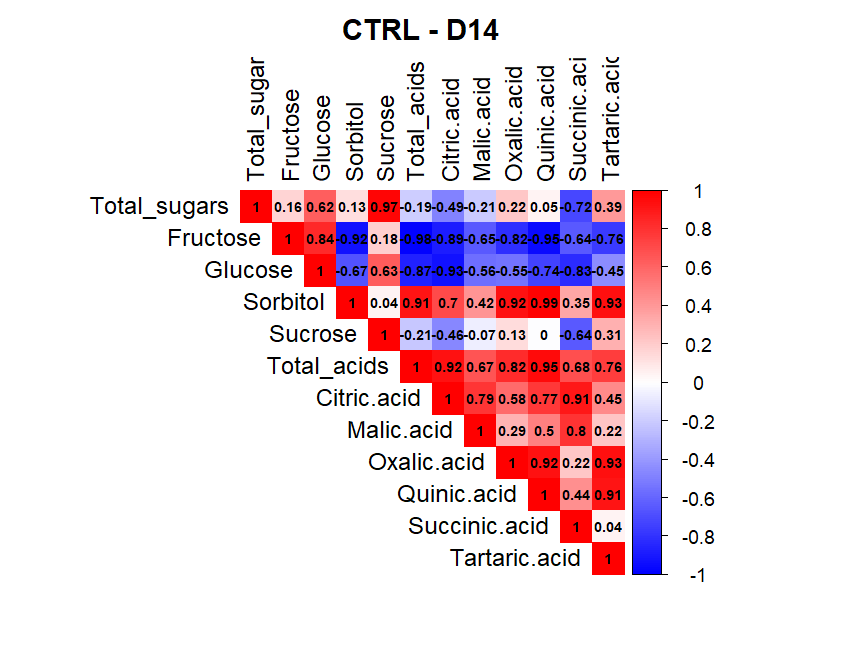

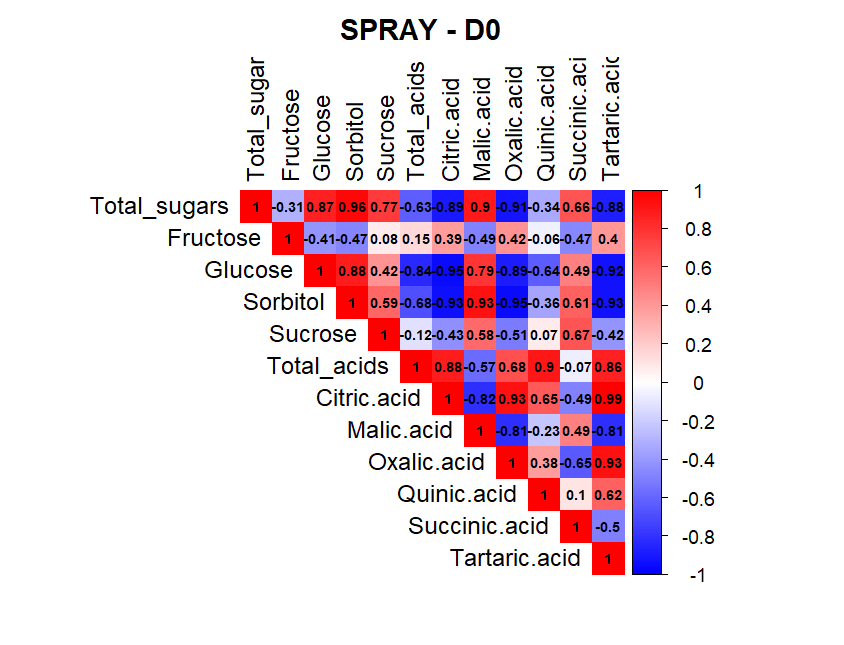

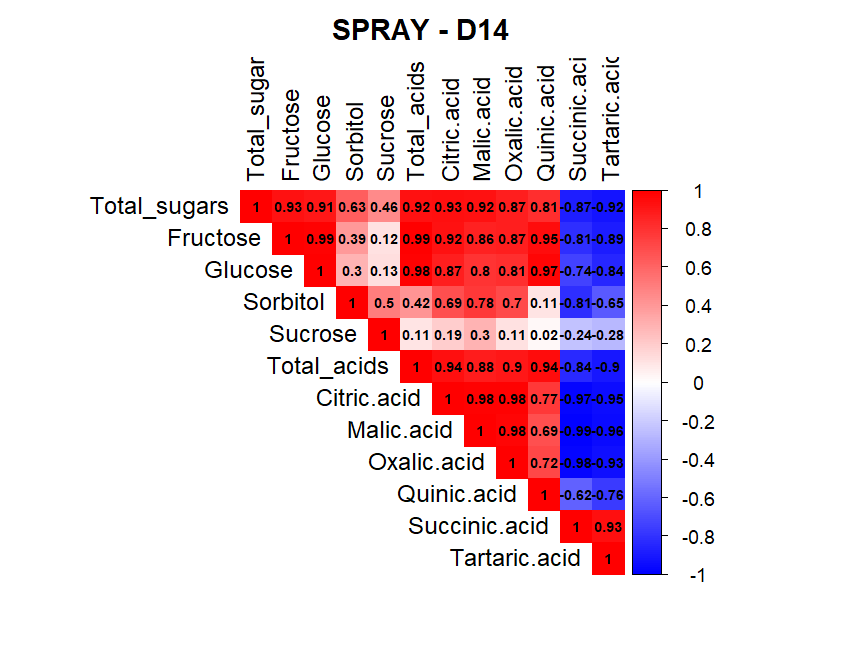


A1

B1

B

A

**Figure S 3.** Correlation matrix between sugar and acid components of ‘Sweet Saretta’ sweet cherries grown in a lowland environment at the day of the harvest (D0) and the end of the shelf life (D14).
